# RETRACTED: Ahmad et al. Deciphering the Potential Neuroprotective Effects of Luteolin Against Aβ_1_–_42_-Induced Alzheimer’s Disease. *Int. J. Mol. Sci.* 2021, *22*, 9583

**DOI:** 10.3390/ijms27146230

**Published:** 2026-07-13

**Authors:** Sareer Ahmad, Myeung Hoon Jo, Muhammad Ikram, Amjad Khan, Myeong Ok Kim

**Affiliations:** Division of Life Science and Applied Life Science (BK21 Four), College of Natural Sciences, Gyeongsang National University, Jinju 52828, Republic of Korea; sareer_50@gnu.ac.kr (S.A.); audgns1217@gnu.ac.kr (M.H.J.); qazafi417@gmail.com (M.I.); amjadkhan1901@gmail.com (A.K.)

The journal retracts the article titled “Deciphering the Potential Neuroprotective Effects of Luteolin against Aβ_1_–_42_-Induced Alzheimer’s Disease” [1], cited above.

Following publication, concerns were raised with the Editorial Office regarding the integrity of the figures presented in this publication [1].

In accordance with the journal’s standard procedures, the Editorial Office and Editorial Board conducted an investigation that identified indications of inappropriate editing and duplication in Figures 4 and 5. Although the authors cooperated during the investigation, explanations and supporting materials provided were not deemed sufficient by the Editorial Board to resolve the identified concerns. Consequently, the Editorial Board has lost confidence in the reliability of the findings and decided to retract this publication [1], as per MDPI’s retraction policy.

This retraction was approved by the Editor-in-Chief of the *IJMS* journal.

The authors have been informed of this retraction and have indicated their disagreement with this decision.
